# Prognostic value of the pretreatment Naples prognostic score in patients with colorectal cancer: a systematic review and meta-analysis

**DOI:** 10.3389/fonc.2024.1498854

**Published:** 2025-01-07

**Authors:** Hui Liu, Dailiang Zhu, Dequan Jiang, Huayang Pang, Xiaolian Yang

**Affiliations:** ^1^ Department of Gastrointestinal Surgery, Chongqing University Jiangjin Hospital, Chongqing, China; ^2^ Department of Anorectal Surgery, Chongqing University Jiangjin Hospital, Chongqing, China; ^3^ Department of Gastrointestinal Cancer Center, Chongqing University Cancer Hospital, Chongqing, China

**Keywords:** colorectal cancer, naples prognostic score, overall survival, disease-free survival, meta-analysis

## Abstract

**Background:**

The prognostic significance of the Naples prognostic score (NPS) in colorectal cancer remains uncertain. This study aims to investigate the correlation between the pretreatment NPS and long-term oncological outcomes in patients with colorectal cancer.

**Methods:**

A comprehensive literature search of electronic databases, including PubMed, Embase, and Web of Science, was conducted up to July 1^st^, 2024. The primary outcomes assessed were survival outcomes. Subgroup analysis and sensitivity analysis were performed during the pooled analysis.

**Results:**

Eight studies including 2571 patients were included. The pooled results indicated that patients in the high NPS group exhibited significantly worse overall survival (HR= 2.08 95%CI: 1.74-2.48; P<0.01; I^2^ = 0%) and disease-free survival (HR=2.03; 95%CI: 1.49-2.77; P<0.01; I^2^ = 30%). Notably, the prognostic significance of NPS on both overall survival and disease-free survival was consistent across different geographical regions, tumor stages, and primary treatments examined in this study. Furthermore, sensitivity analyses confirmed the robustness of these combined results.

**Conclusion:**

The pretreatment NPS could serve as a valuable biomarker for predicting long-term oncological outcomes in patients diagnosed with colorectal cancer.

## 1 Background

Colorectal cancer (CRC) continues to be the third most commonly diagnosed cancer and the second leading cause of cancer-related deaths worldwide (1). Despite advancements in surgical techniques, targeted therapy, and immunotherapy for CRC patients, the clinical prognosis remains unsatisfactory. Currently, prognostic predictions for CRC primarily rely on the AJCC TNM staging system; however, long-term survival can vary even among patients with the same TNM stage (2). In order to enhance the long-term survival of patients with CRC, the utilization of robust prognostic biomarkers capable of identifying high-risk individuals could yield significant clinical benefits for tailoring individual postoperative follow-up plans and treatment strategies.

​ According to emerging evidence, the immune and nutritional status of hosts play a pivotal role in the progression and survival of cancer patients (3). Building upon these insights, several inflammation/nutrition indicators have been developed to forecast survival outcomes for cancer patients, including serum albumin (4), neutrophil-to-lymphocyte ratio (NLR) (5), and lymphocyte-monocyte ratio (LMR) (6). However, these biomarkers fall short in their capacity to accurately predict prognosis. The Naples prognostic score (NPS), which is calculated based on peripheral albumin level, total cholesterol level, NLR, and LMR, was therefore developed as a prognostic assessment tool for CRC patients undergoing surgery by Galizia et al. (7) in 2017. Subsequently, an increasing number of studies have investigated the association between the NPS and clinical outcomes in various malignancies (8–10).

To the best of our knowledge, no meta-analysis has been conducted to investigate the prognostic value of NPS in patients with CRC. Therefore, we undertook a meta-analysis to explore the association between the pretreatment NPS and long-term outcomes in CRC patients.

## 2 Methods

### 2.1 Search strategy

The present systematic review and meta-analysis was conducted according to the Preferred Reporting Items for Systematic Reviews and Meta-Analyses (PRISMA) guidelines (11). Relevant studies from PubMed, Embase and Web of Science were examined up to July 1^st^, 2024. The following combination of key words was used to search the related studies: (“Naples prognostic score”) AND (((colorectal) or (colon) or (rectum) or(rectal)) and ((cancer) or (cancers) or (tumor) or (tumors)or (carcinoma))). Language restrictions were not applied during the search process. Additionally, the references of the included studies were thoroughly scanned for supplementary reports. The search was independently performed by two investigators (H-L and HY-P).

### 2.2 Study selection

The inclusion criteria were as follows: (1) Studies investigating the association between the pretreatment NPS and survival outcomes in patients with colorectal cancer; (2) Hazard ratio (HR) along with a 95% confidence interval (CI) was either directly reported or could be calculated; (3) The specific cut-off value of NPS was clearly stated. The exclusion criteria were as follows: (1) Studies lacking separate data for colorectal cancer patients; (2) Case reports, reviews, conference papers, and letters were excluded; (3) Duplicate data.

### 2.3 Data extraction and quality assessment

Two reviewers (H-L and HY-P) conducted the data extraction independently and cross-checked all the results. The extracted data encompassed essential information such as the first author, publication year, study interval, country, study design and sample size, cut-off value, clinicopathological features including age, sex, ASA (American Society of Anesthesiologists) score, BMI (Body mass index), tumor size, tumor differentiation, lymphovascular invasion, perineural invasion and tumor stage, as well as survival outcomes.

The quality assessment of included studies was performed using the Newcastle-Ottawa Scale (NOS) (12), which consists of predefined eight items. Each study received a final score ranging from 0 to 9 after thorough evaluation; scores between 7-9 were considered indicative of high-quality research.

### 2.4 Outcomes assessment

In this study, the primary outcomes focused on survival measures, specifically overall survival (OS), disease-free survival (DFS), and progression-free survival (PFS). The second outcome was to assess the correlation between the pretreatment NPS and clinicopathological features of colorectal cancer. It is worth noting that due to DFS and PFS shared similar endpoints, they were analyzed collectively as a single outcome measure (DFS), in accordance with previous recommendations (13, 14).

### 2.5 Statistical analysis

The mean difference (MD), risk ratio (RR), and HR along with their corresponding 95% CIs were utilized as the effect size for continuous variables, dichotomous variables and survival outcomes, respectively. For studies that reported median with range or inter-quartile range, data were converted into mean with standard deviation (SD) using the method reported by McGrath et al. (15). In cases where survival data were not directly reported in the literature, we extracted them from the survival curves using the methods described by Tierney et al. (16). Statistical heterogeneity among the included studies was assessed using I^2^ statistics. Random-effects models were employed to calculate effect sizes during the meta-analysis. Subgroup analysis and sensitivity analysis were conducted to evaluate the credibility of pooled results. Begg’s funnel plot was used to assess potential publication bias. A two-tailed P value <0.05 was considered statistically significant. All statistical analyses were performed using R software, version 4.2.1.

## 3 Results

### 3.1 Study characteristics

**Figure 1:**
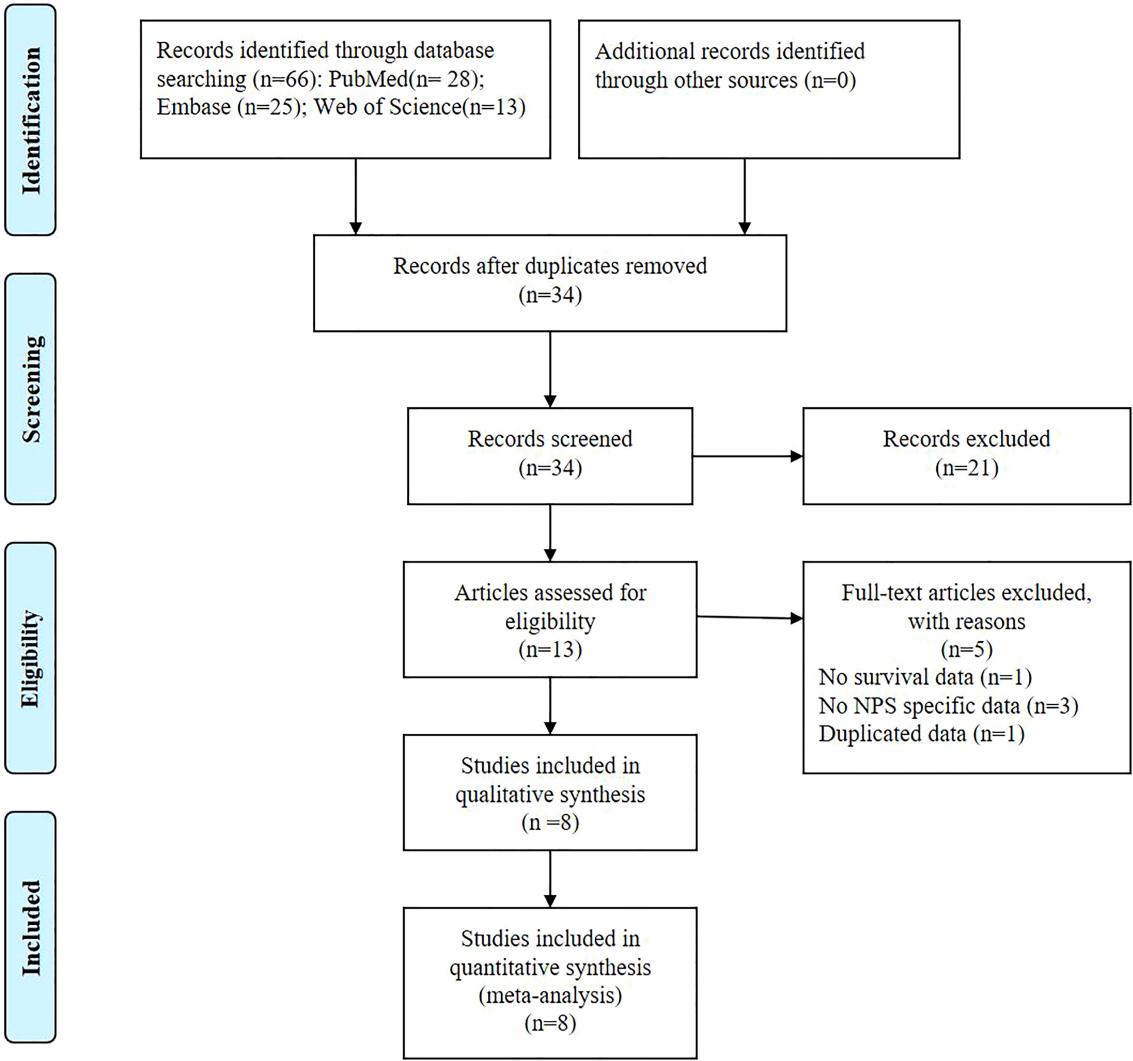
The PRISMA Flowchart of study selection.

The databases yielded a total of 66 records, as depicted in **Figure 1**. After assessment of titles, abstracts, and full texts, eight studies (7, 17–23) were included in the present study. **Tables 1** and **2** provided a summary of the basic information and clinical characteristics of these included studies, respectively. This study encompassed a total of 2571 patients from China, Japan, Korea, and Italy. The publication years ranged from 2017 to 2023 with sample sizes varying between 136 and 533 individuals. Among the included studies, seven focused on colorectal cancer while one specifically examined rectal cancer. Regarding primary treatment modalities, six studies involved surgery while neoadjuvant chemoradiotherapy was employed in one study and first-line chemotherapy in another study. All included studies evaluated OS, six assessed DFS, and one evaluated PFS. Notably, these studies demonstrated good quality with scores ranging from six to seven (**Table 2**, **Supplementary Table S1**).

**Table 1:** Basic information of included studies.

Reference	Country	Study interval	Study design	Sample size	Age, years (Median/Mean)	Sex (Male: Female)	Time of blood collection	Exclusion of diseases affecting serum biomarkers
Galizia, 2017 (7)	Italy	2004-2014	Retrospective	562	NA	228:334	One week before surgery	Yes
Gu, 2023 (17)	China	2014-2018	Retrospective	196	65	102:94	Two weeks before surgery	Yes
Lieto, 2023 (18)	Italy	2014-2021	Retrospective	403	NA	207:196	Before surgery	No
Miyamoto, 2023 (19)	Japan	2005-2019	Retrospective	272	63	141:131	Two weeks before chemotherapy	Yes
Park, 2023 (20)	Korea	2005-2012	Retrospective	164	NA	95:69	Before surgery	No
Pian, 2022 (21)	China	2010-2015	Retrospective	305	63	183:122	Two weeks before chemotherapy	No
Sugimoto, 2023 (22)	Japan	2008-2016	Retrospective	533	70	313:220	Before surgery	No
Zhu, 2021 (23)	China	2015-2020	Retrospective	136	59.2	68:68	Before chemoradiotherapy	No

**Table 2:** Clinicopathological characteristics of included studies.

Reference	Cut-off value of NPS	Tumor location	Primary treatment	TNM stage	Median follow-up time, months	Survival outcomes	Multivariate analysis	NOS
Galizia, 2017 (7)	Albumin: 4.0 mg/dL, Total cholesterol level: 180 mg/dL, NLR: 2.96, LMR: 4.44	Colorectal cancer	Surgery	I-IV	34.7	OS; DFS	Yes; Yes	7
Gu, 2023 (17)	Albumin: 3.63 mg/dL, Total cholesterol level: 3.48 mmol/dL, NLR: 5.33, LMR: 3.19	Colorectal cancer	Surgery	I-IV	NA	OS; DFS	Yes; Yes	7
Lieto, 2023 (18)	Albumin: 4.0 mg/dL, Total cholesterol level: 180 mg/dL, NLR: 2.96, LMR: 4.44	Colorectal cancer	Surgery	0-IV	NA	OS; DFS	Yes; Yes	6
Miyamoto, 2023 (19)	Albumin: 4.0 mg/dL, Total cholesterol level: 180 mg/dL, NLR: 2.96, LMR: 4.44	Colorectal cancer	First line chemotherapy	IV	30.1	OS	Yes	6
Park, 2023 (20)	Albumin: 4.0 mg/dL, Total cholesterol level: 180 mg/dL, NLR: 2.96, LMR: 4.44	Colorectal cancer	Surgery	II-III	NA	OS; DFS	No; No	6
Pian, 2022 (21)	Albumin: 4.0 mg/dL, Total cholesterol level: 180 mg/dL, NLR: 2.96, LMR: 4.44	Colorectal cancer	Surgery	I	87	OS; DFS	No; No	7
Sugimoto, 2023 (22)	Albumin: 4.0 mg/dL, Total cholesterol level: 180 mg/dL, NLR: 2.96, LMR: 4.44	Colorectal cancer	Surgery	I-III	63	OS; DFS	Yes; Yes	6
Zhu, 2021 (23)	Albumin: 4.0 mg/dL, Total cholesterol level: 180 mg/dL, NLR: 2.96, LMR: 4.44	Rectal cancer	Neoadjuvant chemo-radiotherapy	II-III	46	OS; PFS	Yes; Yes	6

NPS, Naples prognostic score; NLR, Neutrophil-to-lymphocyte ratio; LMR, Lymphocyte-monocyte ratio; OS, Overall survival; DFS, Disease-free survival; PFS, Progression-free survival; NA, Not available; NOS, Newcastle-Ottawa Scale.

### 3.2 Relationship between the pretreatment NPS and OS

**Figure 2:**
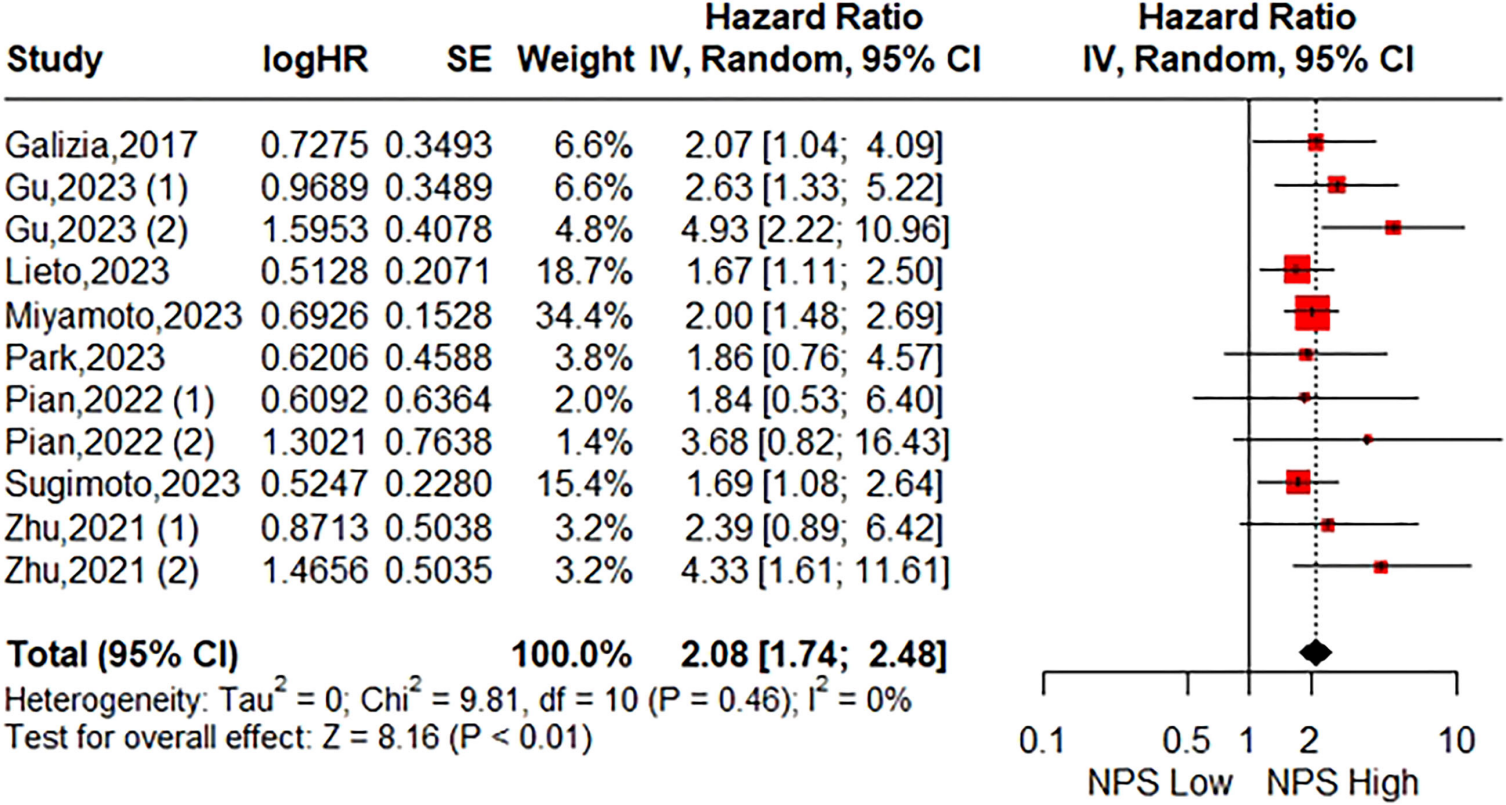
Forest plot assessing the relationship between the pretreatment NPS and OS.

The association between the NPS and OS was investigated in eight studies involving 2571 patients. The pooled HR was 2.08 (95%CI: 1.74-2.48; P<0.01), indicating a significant correlation between high NPS and worse OS in CRC patients (**Figure 2**). Furthermore, subgroup analyses based on country, time of blood examination, exclusion of diseases effecting serum biomarkers, category of NPS, definition of NPS, tumor location, TNM stage, primary treatment, Multivariate analysis, and NOS were performed. As shown in **Table 3** and **Supplementary Figure S1**, the results from all subgroup analyses consistently demonstrated that patients with high NPS had significantly reduced OS compared to those with low NPS group. Additionally, sensitivity analysis by omitting one study at a time showed no significant change in the overall outcome (**Supplementary Figure S3A**).

**Table 3:** Results of subgroup analyses of overall survival.

Variables	Subgroups	Studies, n	Patients, n	HR (95%CI)	I^2^ (%)
	Total	8	2571	2.08 (1.74-2.48)	0
Country	China	3	637	1.85 (1.52-2.26)	0
	Others	5	1934	3.19 (2.18-4.67)	0
Time of blood collection	Within 1 week	1	562	2.07 (1.04-4.09)	–
	Within 2 weeks	3	773	2.56 (1.73-3.77)	20
	Unknown	4	1236	1.86 (1.43-2.42)	0
Exclusion of diseases affecting serum biomarkers	Yes	3	1030	2.42 (1.72-3.39)	34
	No	5	1541	1.89 (1.47-2.44)	0
Definition of NPS	Standard	7	2375	1.95 (1.62-2.35)	0
	Modified	1	196	3.48 (1.89-6.40)	27
Category of NPS	NPS 1 vs. 0	3	637	2.42 (1.45-4.03)	0
	NPS 2 vs. 0	3	637	4.52 (2.55-8.02)	0
	High vs. Low	5	1934	1.85 (1.52-2.26)	0
Tumor location	Colorectal cancer	7	2435	2.02 (1.68-2.42)	0
	Rectal cancer	1	136	3.22 (1.60-6.47)	0
TNM stage	Nonmetastatic	4	1138	2.06 (1.48-2.87)	0
	Mixed	3	1161	2.39 (1.54-3.70)	50
	Metastatic	1	272	2.00 (1.48-2.69)	–
Primary treatment	Neoadjuvant	1	136	3.22 (1.60-6.47)	7
	Surgery	6	2163	2.08 (1.61-2.68)	–
	Systematic	1	272	2.00 (1.48-2.69)	0
Multivariate analysis	Yes	6	2102	2.07 (1.73-2.49)	24
	No	2	469	2.11 (1.10-4.07)	0
NOS	6	4	1563	2.05 (1.46-2.87)	0
	7	4	1008	2.11 (1.71-2.62)	31

NPS, Naples prognostic score; NOS, Newcastle-Ottawa Scale; HR, Hazard ratio; CI, Confidence interval.

### 3.3 Relationship between the pretreatment NPS and DFS

**Figure 3:**
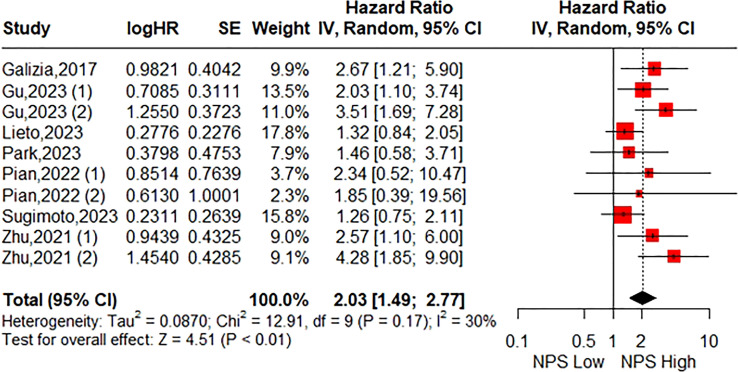
Forest plot accessing the relationship between the pretreatment NPS and DFS.

A total of seven studies consisting of 2299 patients reported on DFS. The pooled HR was 2.03 (95%CI: 1.49-2.77; P<0.01; I^2^ = 30%), indicating that a significant association between high NPS group and poorer DFS compared to the low NPS group (**Figure 3**). Similarly, Stratification by the same parameters based on the aforementioned parameters revealed that the incorporated results were almost consistent in each subgroup (**Table 4**, **Supplementary Figure S2**). Sensitivity analysis confirmed the stability of the pooled result (**Supplementary Figure S3B**).

**Table 4:** Results of subgroup analyses of disease-free survival.

Variables	Subgroups	Studies, n	Patients, n	HR (95%CI)	I^2^ (%)
	Total	7	2299	2.03 (1.49-2.77)	30
Country	China	3	637	2.75 (1.93-3.91)	0
	Others	4	1662	1.45 (1.08-1.94)	0
Time of blood collection	Within 1 week	1	562	2.67 (1.21-5.90)	–
	Within 2 weeks	2	501	2.49 (1.61-3.84)	0
	Unknown	4	1236	1.78 (1.15-2.76)	50
Exclusion of diseases affecting serum biomarkers	Yes	2	758	2.57 (1.72-3.85)	0
	No	5	1541	1.79 (1.21-2.64)	28
Definition of NPS	Standard	6	2103	1.88 (1.32-2.70)	27
	Modified	1	196	2.57 (1.51-4.37)	21
Category of NPS	1 vs. 0	3	637	2.21 (1.38-3.54)	0
	2 vs. 0	3	637	3.62 (2.13-6.16)	0
	Others	4	1662	1.45 (1.08-1.94)	0
Tumor location	Colorectal cancer	6	2163	1.80 (1.32-2.45)	14
	Rectal cancer	1	136	3.32 (1.83-6.04)	0
TNM stage	Nonmetastatic	4	1138	2.02 (1.25-3.26)	27
	Mixed	3	1161	2.08 (1.34-3.25)	50
Primary treatment	Neoadjuvant	1	136	3.32 (1.83-6.04)	0
	Surgery	6	2163	1.80 (1.32-2.45)	14
Multivariate analysis	Yes	5	1830	2.12 (1.48-3.04)	52
	No	2	469	1.69 (0.81-3.52)	0
NOS	6	4	1563	2.02 (1.26-3.22)	28
	7	3	736	2.09 (1.33-3.30)	49

NPS, Naples prognostic score; NOS, Newcastle-Ottawa Scale. HR, Hazard ratio; CI, Confidence interval.

### 3.4 Relationship between the pretreatment NPS and clinicopathological factors

Altogether, four studies with 1029 patients reported a relationship between the pretreatment NPS and clinicopathological factors of colorectal cancer. As shown in **Table 5** and **Supplementary Figure S4**, an increased NPS was markedly related to higher age (MD=-4.89; 95%CI: -9.13 to -0.64; I^2^ = 80%), lower BMI (MD=0.52; 95%CI: 0.02 to 1.01; I^2^ = 0%), and higher ASA score (RR=0.52; 95%CI: 0.36 to 0.75; I^2^ = 30%). However, NPS was not significantly associated with sex, tumor size, tumor differentiation, TNM stage, lymphovascular invasion and perineural invasion.

**Table 5:** The correlation between the pretreatment NPS and clinicopathological factors of colorectal cancer.

Clinicopathological factors	Studies, n	Patients, n	RR/MD (95%CI)	I^2^(%)
Age, years (Mean ± SD)	3	865	-4.89 (-9.13 to -0.64)	80
Sex (Male)	4	1029	1.08 (0.82-1.42)	66
BMI, kg/m^2^ (Mean ± SD)	3	893	0.52 (0.02-1.01)	0
ASA score (≥III)	3	893	0.52 (0.36-0.75)	30
Tumor size, cm (Mean ± SD)	2	300	-0.49 (-2.15-1.18)	0
Tumor differentiation (Poor)	4	1029	0.66 (0.36-1.20)	51
TNM stage (III/IV)	4	1029	0.95 (0.75-1.21)	49
Lymphovascular invasion (Yes)	3	496	0.80 (0.56-1.12)	0
Perineural invasion (Yes)	3	496	0.89 (0.42-1.90)	63

RR, Risk ratio; MD, Mean difference; CI, Confidence interval; SD, Standard deviation; BMI, Body mass index; ASA, American Society of Anesthesiologists.

### 3.5 Publication bias

**Figure 4:**
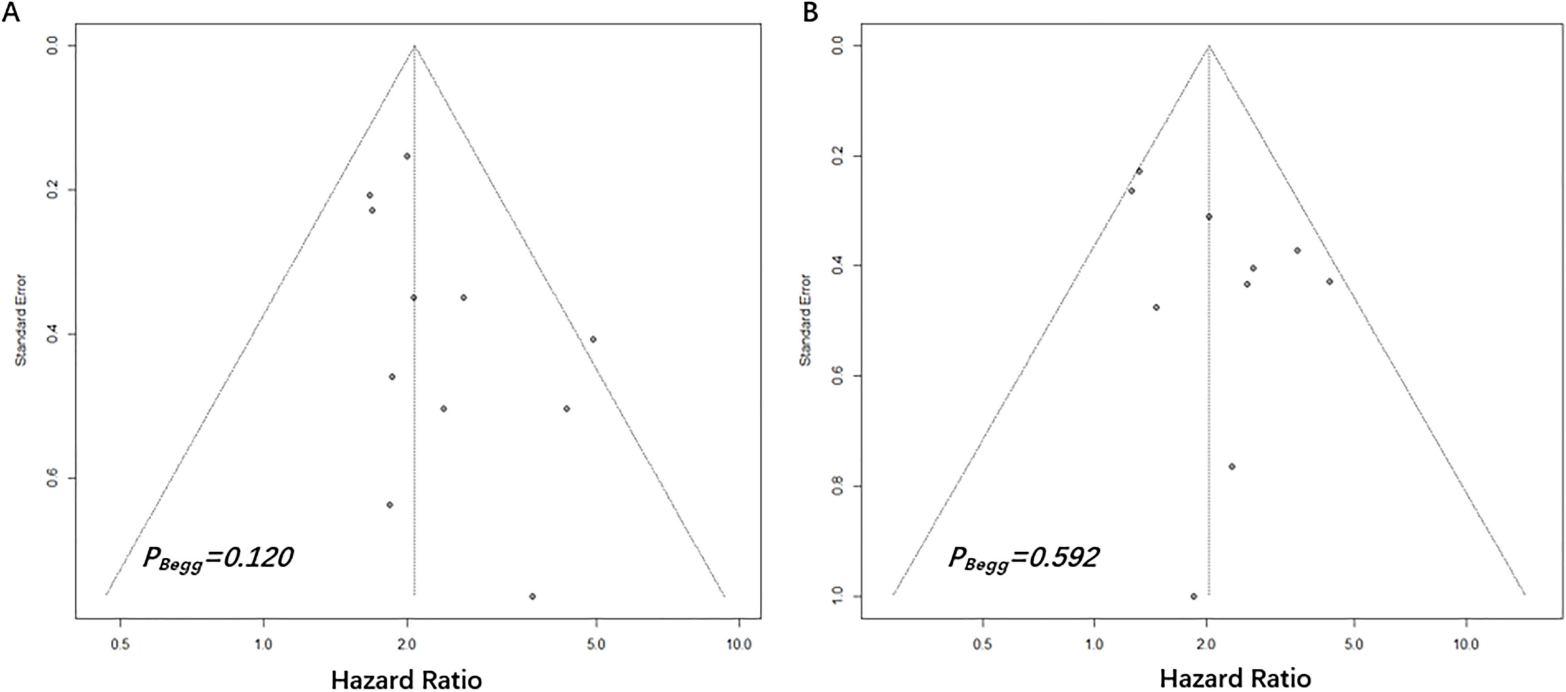
Begg’s funnel plots assessing publication bias between pretreatment NPS and OS **(A)** and DFS **(B)**. The Begg’s P values were 0.120 and 0.592, respectively.

The Begg’s funnel plots were presented in **Figure 4**. According to the results of Begg’s test, no significant publication bias was observed in the current study regarding the association between NPS and OS (P=0.120) as well as DFS (P=0.592).

## 4 Discussion

Uncontrolled inflammation and malnutrition are significant characteristics observed in cancer patients, which can result in a suboptimal response to medical treatment and deteriorating long-term outcomes (24, 25). Currently, there is substantial evidence confirming the utility of peripheral blood-based parameters as valuable biomarkers reflecting the inflammatory and nutritional status of cancer patients (26). The minimally invasive retrieval, cost-effectiveness, and objectivity associated with complete blood count-based biomarkers make them highly appealing to clinicians. Consequently, an increasing number of studies are focused on establishing clinically useful biomarkers.

In this context, the NPS was established as a potential tool to make clinical prognostic evaluation using four peripheral blood parameters: albumin level, total cholesterol level, NLR and LMR (7). Since then, the NPS has gradually gained popularity in assessing the prognosis of various cancers due to its easy availability and convenient calculation (27–29). Wang et al. (30) conducted a meta-analysis of seven studies and reported that elevated NPS levels are associated with poorer OS and DFS in patients with lung cancer. Another meta-analysis by Guo et al. (31) also confirmed the practical prognostic value of NPS in predicting the prognosis of esophageal cancer. Moreover, the clinical significance of NPS has been validated in other malignancies, including gastric cancer (27) and hepatocellular carcinoma (32). However, considering the heterogeneity among different types of cancers, it is crucial to investigate the applicability of NPS in colorectal cancer.

We conducted an extensive literature search and identified eight studies involving 2571 patients with CRC. Our pooled analyses revealed that patients in the high NPS group had a 2.08-fold increased risk of poor OS and a 2.03-fold increased risk of poor DFS compared to those with low NPS. By including a diverse range of patients from different ethnic backgrounds, disease stages, cut-off values of NPS and treatment options, we were able to investigate the utility of the pretreatment NPS as a screening metric for predicting survival outcomes in CRC patients. Subgroup analyses consistently demonstrated that the NPS could be considered an independent prognostic biomarker in CRC patients. Furthermore, our sensitivity analyses confirmed the robustness of these clinical findings, while Begg’s tests provided no evidence of publication bias. These results enhance the credibility of our conclusions.

The high discriminatory value of the NPS can be attributed to its combined utilization of various markers related to nutrition and inflammation, including albumin, cholesterol, neutrophils, monocytes, and lymphocytes. Firstly, albumin is a widely recognized indicator that reflects a patient’s nutritional status. Hypoalbuminemia has been shown to be significantly associated with poor wound healing, increased risk of infections, and reduced survival in cancer patients (33, 34). Additionally, serum albumin plays a crucial role in inhibiting the production of pro-inflammatory cytokines and enhancing cell-mediated immunity (35). Secondly, cholesterol is an essential component of cell membranes and plays a vital role in maintaining cellular function. Low levels of cholesterol have been suggested to promote tumor progression and worsen patient prognosis in various cancers (36, 37). ​This may be due to the requirement for cholesterol consumption by tumors for growth (38). Thirdly, as mature markers associated with inflammation, NLR and LMR have been extensively validated as predictors of short- and long-term adverse outcomes in malignancies (5, 6). The underlying mechanism involves neutrophils creating a favorable microenvironment for tumor cell proliferation while promoting tumor cell progression and invasion (39). Monocytes, differentiated into tumor-associated macrophages (TAMs), can induce apoptosis of T cells with antitumor functions and stimulate tumor angiogenesis (40, 41). Lymphocytes, particularly CD3+ and CD8+ T cells, in the inhibition of tumor cell growth through induction of tumor cell lysis and apoptosis have been well-documented, along with their association with improved long-term survival in cancer patients (42). Despite certain subgroups such as regulatory T cells and T helper 17 cells playing a pro-cancer role and being linked to poor prognosis, numerous clinical studies have consistently demonstrated a positive correlation between total lymphocyte count and cancer prognosis (42, 43).

The present meta-analysis had several limitations. Firstly, all of these studies were retrospective in nature, which may introduce selection bias and necessitate further investigation through prospective studies. Secondly, the majority of included studies originated from Asian countries, potentially limiting the generalizability of the NPS to Western populations. Thirdly, microsatellite instability (MSI) and microsatellite stability (MSS) colorectal cancer exhibit distinct characteristics in terms of their immune microenvironment. Although Lieto et al. (18) reported that preoperative NPS was an independent prognostic factor for overall survival in colorectal cancer regardless of the MSI status, further studies are still required to validate the prognostic significance of NPS in both subtypes. Lastly, the vast majority of included patients underwent surgery, therefore, the predictive value of NPS in the neoadjuvant therapy, first-line treatment, and even in later-line treatment still need to be further explored.

## 5 Conclusion

Our findings suggest that the pretreatment NPS may serve as a valuable prognostic biomarker for patients diagnosed with colorectal cancer, as those in the high NPS group exhibit poorer overall survival and disease-free survival rates. Clinicians can utilize this informative indicator to stratify patients and develop personalized treatment plans. However, further research is necessary to validate the utility of this index in colorectal cancer.

## Data Availability

The original contributions presented in the study are included in the article/**Supplementary Material**. Further inquiries can be directed to the corresponding author/s.
