# Supplementary material for: Prognostic value of the pretreatment Naples prognostic score in patients with colorectal cancer: a systematic review and meta-analysis
Source: Front Oncol. 2025 Jan 7;14:1498854. doi: 10.3389/fonc.2024.1498854 (PMC11746047; doi:10.3389/fonc.2024.1498854)
Supplement: Supplementary file 1 [file DataSheet1.docx]

Table S1. Quality assessment of included studies using Newcastle-Ottawa Scale.

| **Cohort study** | **Representatives of the exposed cohort** | **Selection of the non-exposed cohort** | **Ascertainment of exposure** | **Was outcome of interest present at start of study** | **Comparability of cohorts on the basis of the design or analysis** | **Assessment of outcome** | **Was follow-up long enough for outcomes to occur** | **Adequate follow up** | **Total** |
| --- | --- | --- | --- | --- | --- | --- | --- | --- | --- |
| Galizia,2017 | 1 | 1 | 1 | 0 | 1 | 1 | 1 | 1 | 7 |
| Gu,2023 | 1 | 1 | 1 | 0 | 1 | 1 | 1 | 1 | 7 |
| Lieto,2023 | 1 | 1 | 1 | 0 | 1 | 1 | 0 | 1 | 6 |
| Miyamoto,2023 | 1 | 1 | 1 | 0 | 1 | 1 | 1 | 1 | 7 |
| Park,2023 | 1 | 1 | 1 | 0 | 0 | 1 | 1 | 1 | 6 |
| Pian,2022 | 1 | 1 | 1 | 0 | 0 | 1 | 1 | 1 | 6 |
| Sugimoto,2023 | 1 | 1 | 1 | 0 | 1 | 1 | 1 | 1 | 7 |
| Zhu,2021 | 1 | 1 | 1 | 0 | 0 | 1 | 1 | 1 | 6 |


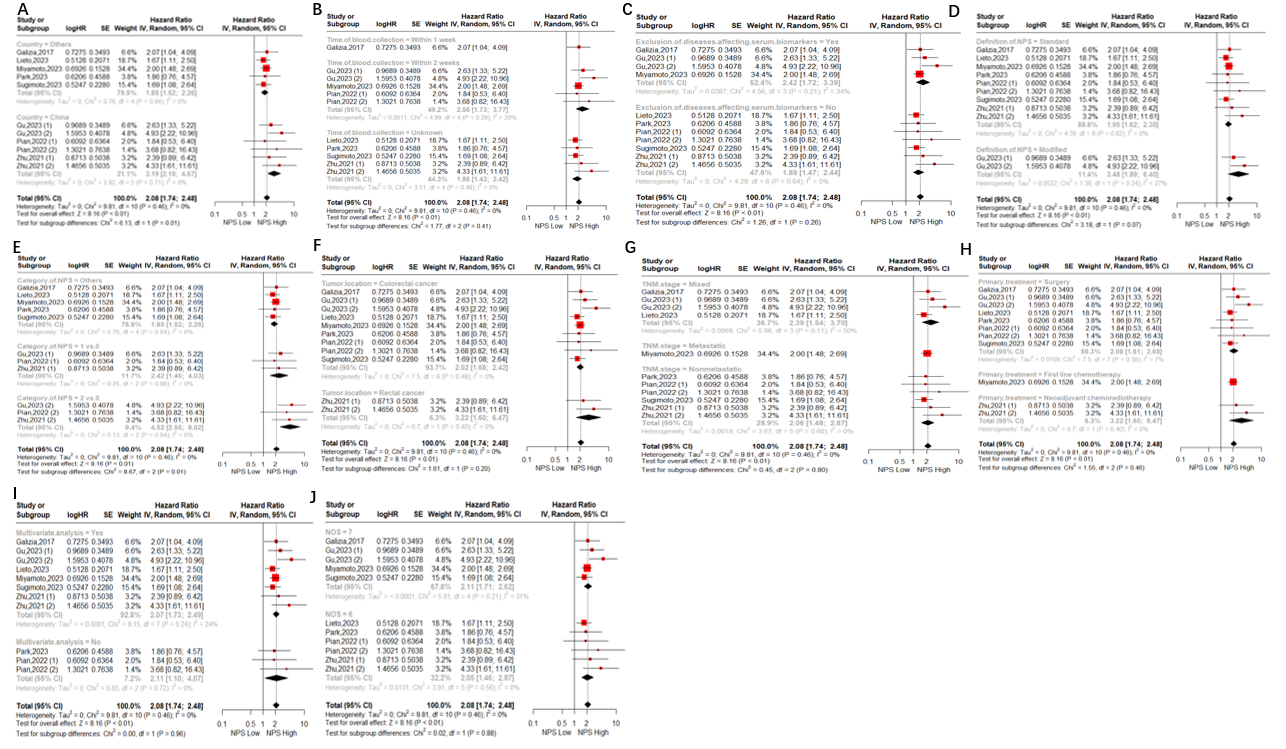


Figure S1. Forest plots of subgroup analyses assessing the relationship between NPS and OS. A: Country (China vs. Others); B: Time of blood collection (Within one week vs. Within two weeks vs. Unknown); C: Exclusion of diseases affecting serum biomarkers (Yes vs. No); D: Definition of NPS (Standard vs. Modified); E: Category of NPS (NPS 1vs. 0; NPS 2 vs. 0; NPS high vs. low); F: Tumor location (Colorectal cancer vs. Rectal cancer); G: TNM stage (non-metastatic vs. mixed vs. metastatic); H: Primary treatment (Neoadjuvant vs. Surgery vs. Systematic); I: Multivariate analysis (Yes vs. No); J: NOS (7 vs. 6).


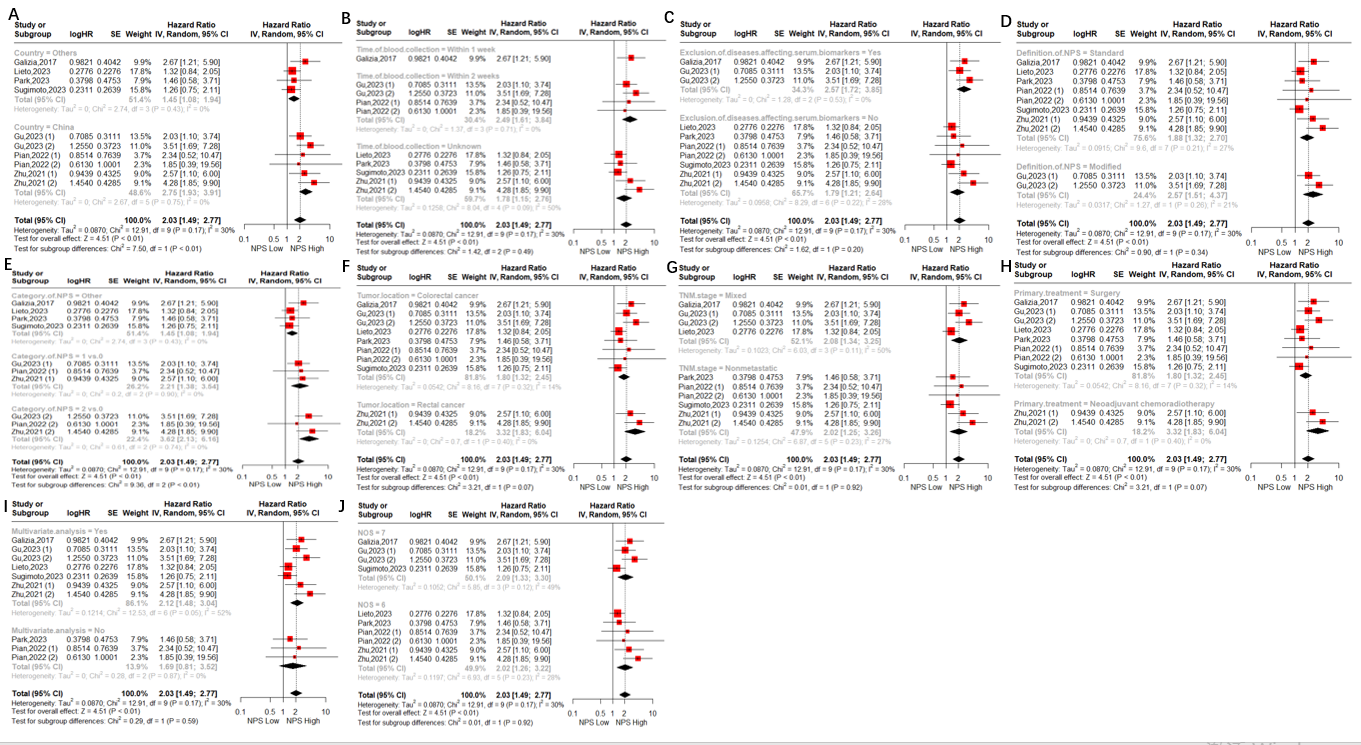


Figure S2. Forest plots of subgroup analyses assessing the relationship between NPS and DFS. A: Country (China vs. Others); B: Time of blood collection (Within one week vs. Within two weeks vs. Unknown); C: Exclusion of diseases affecting serum biomarkers (Yes vs. No); D: Definition of NPS (Standard vs. Modified); E: Category of NPS (NPS 1vs. 0; NPS 2 vs. 0; NPS high vs. low); F: Tumor location (Colorectal cancer vs. Rectal cancer); G: TNM stage (non-metastatic vs. mixed); H: Primary treatment (Neoadjuvant vs. Surgery); I: Multivariate analysis (Yes vs. No); J: NOS (7 vs. 6).


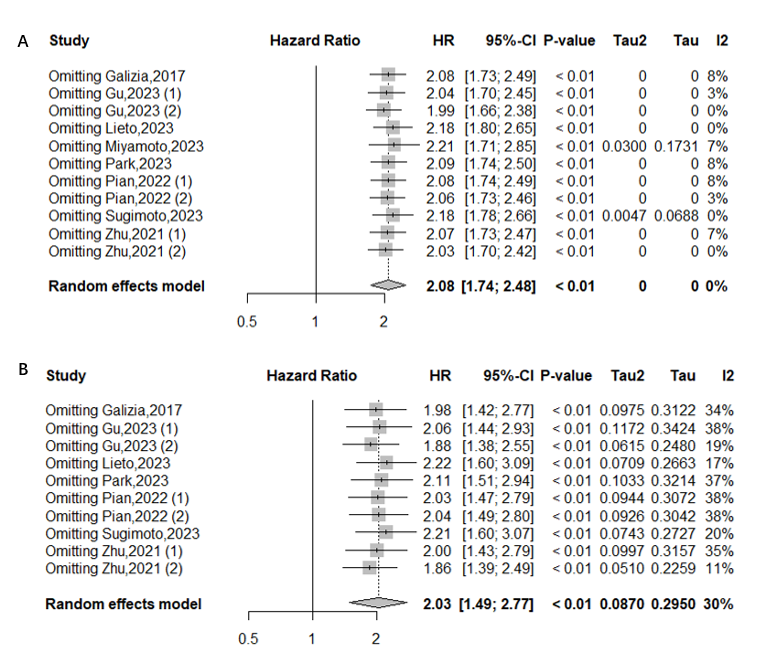


Figure S3. Sensitivity analyses assessing OS (A) and DFS (B).


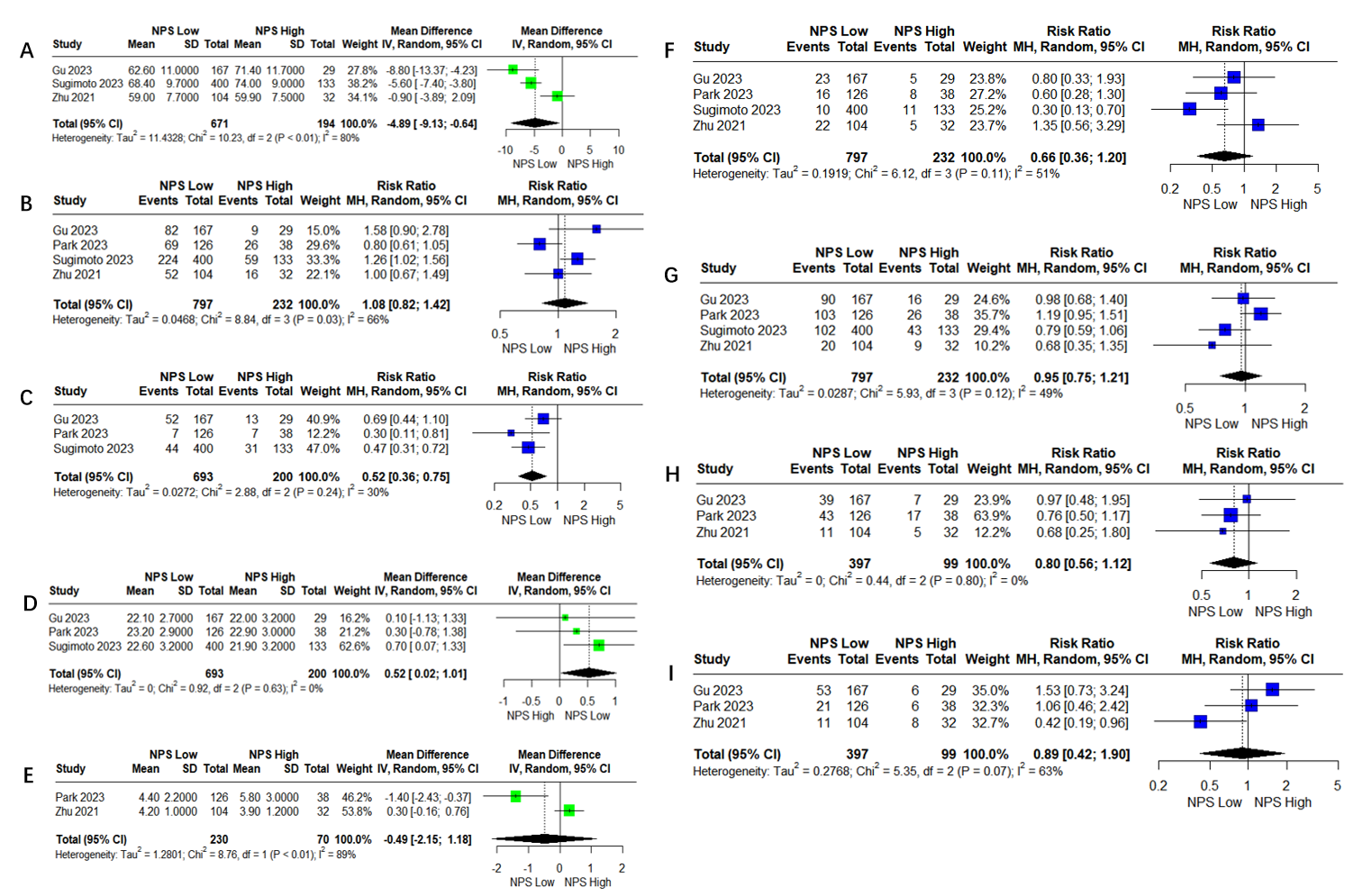


Figure S4. Forest plots assessing the correlation between NPS and clinicopathological factors. A: Age, years; B: Sex (Male); C: ASA score (≥3); D: BMI, kg/m^2^; E: Tumor size, cm; F: Tumor differentiation (Poor) ; G: TNM stage (III/IV); H: Lymphovascular invasion (Yes); I: Perineural invasion (Yes).
